# Linking unfolded protein response to ovarian cancer cell fusion

**DOI:** 10.1186/s12885-022-09648-4

**Published:** 2022-06-07

**Authors:** Lucile Yart, Daniel Bastida-Ruiz, Mathilde Allard, Pierre-Yves Dietrich, Patrick Petignat, Marie Cohen

**Affiliations:** 1 Center for Translational Research in Onco-Hematology, Faculty of Medicine, University of Geneva, Rue Michel Servet 1, CH-1206 Geneva, Switzerland; 2 Department of Pediatrics, Gynecology and Obstetrics, Faculty of Medicine, University of Geneva, Rue Michel Servet 1, CH-1206 Geneva, Switzerland; 3 Present address: Research Center of Cancerology and Immunology Nantes-Angers, Department of Biology, University of Nantes, FR-44035 Nantes, France

**Keywords:** Unfolded protein response, Ovarian cancer, Polyploid giant cancer cell, Cell fusion, Invasion

## Abstract

**Background:**

Polyploid giant cancer cells (PGCCs) have been observed in epithelial ovarian tumors. They can resist antimitotic drugs, thus participating in tumor maintenance and recurrence. Although their origin remains unclear, PGCC formation seems to be enhanced by conditions that trigger the unfolded protein response (UPR) such as hypoxia or chemotherapeutic drugs like paclitaxel. Hypoxia has been shown to promote the formation of ovarian PGCCs by cell fusion. We thus hypothesized that the UPR could be involved in EOC cell fusion, possibly explaining the occurrence of PGCCs and the aggressiveness of EOC.

**Methods:**

The UPR was induced in two ovarian cancer cell lines (SKOV3 and COV318). The UPR activation was assessed by Western blot and polyploidy indexes were calculated. Then, to confirm the implication of cell fusion in PGCC formation, two populations of SKOV3 cells were transfected with plasmids encoding for two distinct nuclear fluorescent proteins (GFP and mCherry) associated with different antibiotic resistance genes, and the two cell populations were mixed in co-culture. The co-culture was submitted to a double-antibiotic selection. The resulting cell population was characterized for its morphology, cyclicity, and proliferative and tumorigenic capacities, in addition to transcriptomic characterization.

**Results:**

We demonstrated that cell fusion could be involved in the generation of ovarian PGCCs and this process was promoted by paclitaxel and the UPR activation. Double-antibiotic treatment of PGCCs led to the selection of a pure population of cells containing both GFP- and mCherry-positive nuclei. Interestingly, after 3 weeks of selection, we observed that these cells were no longer polynucleated but displayed a single nucleus positive for both fluorescent proteins, suggesting that genetic material mixing had occurred. These cells had reinitiated their normal cell cycles, acquired an increased invasive capacity, and could form ovarian tumors *in ovo*.

**Conclusions:**

The UPR activation increased the in vitro formation of PGCCs by cell fusion, with the newly generated cells further acquiring new properties. The UPR modulation in ovarian cancer patients could represent an interesting therapeutic strategy to avoid the formation of PGCCs and therefore limit cancer relapse and drug resistance.

**Supplementary Information:**

The online version contains supplementary material available at 10.1186/s12885-022-09648-4.

## Background

Epithelial ovarian cancer (EOC) is the most common type of ovarian cancer, representing 9 out of 10 tumors [1]. It is the seventh most common cancer in women and the leading cause of gynecologic cancer-related deaths in Western countries [2]. EOC is usually managed by cytoreductive surgery followed by platinum-based chemotherapy with an expected response rate to primary therapy of more than 70% [3]. Nevertheless, 2 years following complete remission, most patients experience cancer recurrence [4], often with platinum-based chemotherapy treatment resistance [3].

The cause of EOC relapses is not completely understood. However, several hypotheses have been proposed. One of them, the cancer stem cell (CSC) theory, is based on the capacity of these cells to generate a proliferative progeny, initiating the growth of a heterogeneous tumor [5]. CSCs present chemotherapy resistance, allowing their survival to the treatment with subsequent proliferation and cancer relapse [6]. The role of CSCs in ovarian cancer was reviewed by Giornelli et al., highlighting the importance of these cells in cancer recurrence and progression [7]. Despite the origin of CSCs remaining elusive, some theories move towards a possible genesis in cancer cell fusion events (reviewed by Lu and Kang [8]). The fusion of cancer cells could give rise to polyploid giant cancer cells (PGCCs) [9], large cells containing several copies of DNA and that are positive for cancer stem cell markers [10]. Interestingly, the presence of PGCCs in ovarian cancer has been reported preferentially at late disease stages and in high pathological grades [10]. We previously showed that primary EOC cells isolated from malignant ascites were able to spontaneously form PGCCs in culture [11]. However, the origin of PGCCs is controversial since they can be formed by fusion or endoreplication processes [8, 9, 12]. Zhang et al. demonstrated that in SKOV3, an EOC cell line established from ascites, the formation of PGCCs was at least partially due to cell fusion events [9]. In their study, the PGCCs derived from EOC cells had striking properties such as different cell cycle regulation processes and division profiles, changing their daughter generation mechanism from mitosis to budding, which confers these cells the resistance to cytotoxic chemotherapy treatments [9]. Additionally, it was found that hypoxia increased PGCCs formation [9, 13–15], but other environments such as chemotherapy could also favor their production [13].

The tumor environment is known to be hypoxic and hypoglycemic due to the low vascularization caused by the fast and erroneous angiogenesis that the tumor mass undergoes (reviewed by Muz et al. [16]). These conditions are capable of triggering the endoplasmic reticulum (ER) stress response or unfolded protein response (UPR) [17] which is essential for cellular adaptation to stressful situations [18]. In homeostatic conditions, the three UPR-associated proteins, protein kinase RNA-like endoplasmic reticulum kinase (PERK), inositol-requiring enzyme 1α (IRE1α), and activating transcription factor 6 (ATF6), remain inactive because of their binding with glucose-regulated protein 78 (GRP78) [reviewed by Bastida-Ruiz et al. [19]]. Under ER stress conditions, unfolded proteins accumulate in the ER lumen, promoting the recruitment of chaperones to enable their correct folding. Among these chaperones, GRP78 leaves the complexes formed with the UPR-associated proteins and induces the UPR activation [20]. This gives rise to a general decrease of protein, by contrast with the increased expression of chaperones and ER-associated protein degradation-related proteins (ERAD) as well as autophagy activation [reviewed by Bastida-Ruiz et al. [19]]. The overexpression of GRP78 serves as a UPR activation marker [21]. GRP78 was shown to be overexpressed in ovarian cancer tissues and correlated with poor patient survival [22], suggesting theUPR has a role in EOC [23]. Interestingly, GRP78 and UPR were involved in trophoblastic cell fusion [24, 25]. We thus hypothesized that the UPR activation could also be involved in EOC cell fusion, possibly explaining the occurrence of PGCCs and the aggressiveness of EOC.

In this article, we confirmed Zhang et al.’s results stating that PGCCs are derived at least partially from cell fusion in SKOV3 cells [9]. We also demonstrated that UPR induces ovarian cancer cell fusion and PGCCs formation. Moreover, we observed that polyploid cells were no longer polynucleated after 3 weeks of antibiotic selection pressure and were able to proliferate in the same manner as their parental cell lines despite having higher DNA content. We then evaluated the advantages that cell fusion could confer to these cells.

## Methods

### Reagents

Chemical hypoxia was induced with Cobalt Chloride (CoCl_2_, Merck, Darmstadt, Germany).

ER stress was induced with HA15 (Selleckchem, Zurich, Switzerland), Thapsigargin (THA, Enzo LifeSciences, Lausen, Switzerland), or Tunicamycin (TUN, Enzo LifeSciences, Lausen, Switzerland).

ER stress was inhibited by 4-(2-aminoethyl) benzenesulfonyl fluoride hydrochloride (AEBSF; Sigma, Darmstadt, Germany), Melatonin (Mel; Sigma, Darmstadt, Germany), STF-083010 (STF; Selleckchem, Zurich, Switzerland), 4μ8C (Selleckchem, Zurich, Switzerland), GSK2656157 (GSK; Selleckchem, Zurich, Switzerland) or Salubrinal (SAL; Tocris Biosciences, Bristol, UK).

### Cancer cell lines and culture

The human ovarian cancer cell lines COV318 (ECACC, Sigma-Aldrich) and SKOV3 (courtesy of Dr. Florence Delie, University of Geneva, Switzerland) were cultured at 37 °C and 5% CO2 in DMEM or RPMI (Gibco, Invitrogen, Basel, Switzerland) respectively, supplemented with 10% fetal bovine serum (FBS, Biochrom AG, Oxoid AG, Basel, Switzerland) and 0.05 mg.mL^− 1^ gentamycin (Invitrogen, Basel, Switzerland).

### Generation of PGCCs by cell fusion

Cytoplasmic staining and FACS sorting: SKOV3 or COV318 cells were stained with either green or far-red cytoplasmic dyes, CellTrace CFSE (green; Life Technologies, Carlsbad, USA), and CellTrace Far-Red (far-red, Life Technologies, Carlsbad, USA), according to the manufacturer’s instructions. For each cell line, 3.5 × 10^5^ green cells and 3.5 × 10^5^ far-red cells were co-seeded immediately after staining in a T175 flask. Twenty-four hours after seeding, cells were treated with 300 μM CoCl_2_ (Merck, Darmstadt, Germany) for 48 h. Cells were then analyzed by flow cytometry with FACS Aria II (Becton Dickinson, Franklin Lakes, USA) for -GFP and far-red signals. Double-positive cells were sorted and seeded in a regular culture medium. Characteristic pictures of the sorted cells were done 16 h after seeding, using a fluorescence microscope (EVOS FL, Life Technologies, Carlsbad, USA).

Transfection and selection: SKOV3 cells were transfected with a pH2B-mCherry-IRES-puro2 plasmid or with pEGFP-CenpA-IRES-neo (Addgene, LGC Standards, Teddington, UK) using JetPEI as the transfection reagent (Polyplus transfection, Illkirch, France) according to the manufacturer’s instructions. Two days after transfection, cells were selected using selection antibiotics (1 μg.mL^− 1^ puromycin, InvivoGen, San Diego, USA for cells transfected with pH2B-mCherry-IRES-puro2 plasmid = SKOV3-Red, and 500 μg.mL^− 1^ G418, Roche Life Science, Penzberg, Germany for cells transfected with pEGFP-CenpA-IRES-neo = SKOV3-Green) for 2 weeks. Cells were then cultured in RPMI medium supplemented with 0.1 μg.mL^− 1^ puromycin for SKOV3-Red cells or with 50 μg.mL^− 1^ G418 for SKOV3-Green cells.

SKOV3-M cell line establishment: SKOV3-Red and SKOV3-Green were mixed (1:1) and cultured for 72 h before adding 0.2 μg.mL^− 1^ puromycin and 100 μg.mL^− 1^ G418 in RPMI. The culture medium was renewed every 2 days.

### Cell treatments

UPR modulators- ER stress was induced in ovarian cancer cells by adding either 100 nM THA, 1 μg.mL^− 1^ TUN or 1 μM HA15 in culture medium. Cells were treated for 48 h before processed for analysis.

UPR pathways were inhibited in ovarian cancer cells by adding either 17.5 μM SAL, 0.3 μM GSK, 8 μM 4μ8C, 2 μM STF, 200 μM AEBSF or 1 mM Mel, in combination with 100 nM THA or 1 μg.mL^− 1^ TUN. Cells were treated for 48 h before processing for analysis.

Paclitaxel treatment- SKOV3 cells were treated with 0, 1, 10, and 100 nM Paclitaxel for 48 h before processing for analysis (immunofluorescence, cell viability and fusion index).

SKOV3-Red and SKOV3-Green (cell ratio 1:1) were treated with 10 nM Paclitaxel for 48 h before changing the culture medium (RPMI, supplemented with 10% FBS and gentamycin) every 2 days for 10 days.

### Zymography

Twenty-four hours after the seeding of SKOV3-M, SKOV3-Red and SKOV3-Green in a 12-well plate, cell culture medium was replaced by a culture medium without FBS and the cells were incubated for 48 h. The supernatants of culture were collected and the proteolytic activity of culture supernatants (*n* = 3) were assayed using gelatin-substrate gel electrophoresis as described previously [26].

### Western blot

Western blot analyses were performed as described by Bastida-Ruiz et al. [24]. Briefly, whole-cell extracts were fractionated by SDS-Page 10% and transferred to nitrocellulose membrane for immunoblot analysis. The membranes were first incubated with the primary antibodies rabbit anti-GRP78 (GL-19, 1∶5000 dilution from Sigma) and mouse anti-GAPDH antibodies (1∶60,000 dilution from Millipore), followed by incubation with the secondary antibodies goat anti-rabbit IgG horseradish peroxidase (HRP)-conjugated (170-6515, 1:3000 dilution, from Bio Rad) and goat anti-mouse IgG HRP-conjugated (sc-2005, 1:3000 dilution, Santa Cruz Biotechnologies).

### Quantification of GFP-positive, mCherry-positive, and double-positive cells

SKOV3-Green, SKOV3-Red, SKOV3-M, and untransfected cells (ct-SKOV3) were resuspended in PBS. Percentages of negative cells, GFP-positive cells, mCherry-positive cells, and GFP and mCherry double-positive cells were measured by flow cytometry analysis (LSR Fortessa, Becton Dickinson, Franklin Lakes, USA). Data were analyzed with FlowJo software (FlowJo LLC, Ashland, USA). This experiment was repeated 3 times.

### Polyploidy index / fusion index

Non-transfected cells: SKOV3 and COV318 cells were fixed in paraformaldehyde (PFA) 4% at 4 °C for 10 min, washed three times in PBS, and stained with Hematoxylin (Sigma, Darmstadt, Germany) for 1 min. Cells were then washed twice with warmed tap water before bright-field imaging was conducted (EVOS, Life Technologies, Carlsbad, USA).

Transfected cells: Cells were seeded either in Nunc Lab-Tek II Chamber slides (Life Technologies, Carlsbad, USA) for confocal microscopy or in μ-Slides 8 Well (Ibidi, Madison, USA) for fluorescent microscopy, and processed for treatment. Cells were then fixed in PFA 4% at 4 °C for 10 min, washed three times in PBS, and incubated in PBS-3% bovine serum albumin (BSA) for 30 min to eliminate non-specific binding. Cells were then stained with Alexa Fluor 647 phalloidin (1∶40 dilution, from Invitrogen, Thermo Scientific) for 20 min at room temperature and rinsed three times in PBS. Nunc Lab-Tek II Chamber slides were then mounted with Vectashield with DAPI (Vector Laboratories, Burlingame, USA) and sealed before imaging with a confocal fluorescent microscope (LSM 800 Airyscan, Zeiss, Iéna, Germany). μ-Slides 8 Well were imaged with a fluorescent microscope (EVOS FL, Life Technologies, Carlsbad, USA). Images were processed using ImageJ freeware.

For both transfected and non-transfected cells, the polyploidy or fusion index expressed in percent was calculated as follows: [(N-S)/T] × 100, where N equals the number of nuclei in syncytia, S equals the number of syncytia and T equals the total number of nuclei counted [27]. This index was calculated for three independent experiments, run in triplicate.

### Cell cycle analysis

SKOV3-Green, SKOV3-Red, and SKOV3-M were resuspended in PBS with 5 μg.mL^− 1^ Hoechst 33342 (Life Technologies, Carlsbad, USA), and incubated for 20 min at 37 °C. The amounts of cells in G0-G1 and G2 phases were then assessed by flow cytometry analysis (LSR Fortessa, Becton Dickinson, Franklin Lakes, USA). Data were analyzed with FlowJo software (FlowJo LLC, Ashland, USA). This experiment was repeated 2 times.

### Cell proliferation

On day 0, SKOV3-Green, SKOV3-Red, and SKOV3-M were resuspended in Hanks’ Balanced Salt Solution (Life Technologies, Carlsbad, USA) and stained with CellTrace Far Red (Life Technologies, Carlsbad, USA) according to the manufacturer’s instructions. The cells were immediately processed for far-red signal analysis by flow cytometry (Gallios, Beckman Coulter, Brea, USA) (D0). Far-red signal dilution was then measured daily for 4 days (D1, D2, D3, and D4). Data were analyzed with FlowJo software (FlowJo LLC, Ashland, USA). This experiment was repeated 3 times.

### Cell viability (MTT assay)

SKOV3, SKOV3-Red, SKOV3-Green, and SKOV3-M cells were seeded in 96-well plates (15′000 cells/well) and incubated for 48 h. The culture medium was then replaced by 100 μL of MTT reagent (1 mg.mL^− 1^) for 2 h. Acidic isopropanol solution (150 μL) was added, and then each well was vigorously mixed to dissolve the precipitated formazan. UV–visible absorption was measured at 540 and 690 nm (as blank). These experiments were carried out in triplicate, three times.

### Invasion assay

Cell invasion assay was performed in an invasion chamber as described elsewhere [28]. Briefly, 1.5 × 10^4^ cells (SKOV3-Red, SKOV3-Green, and SKOV3-M) in 100 μL of RPMI supplemented with 10% FBS and 1% gentamicin were added to the upper compartment of the transwell chambers. RPMI supplemented with 20% FBS and 1% gentamicin (400 μL) was added to the lower chamber for 48 h at 37 °C in a CO_2_ (5%) incubator. After incubation, viable cells that invaded collagen were stained with crystal violet, and measurement was performed at 540 nm. This assay was repeated three times and each experiment was run in triplicate. Data were normalized by proliferation values (MTT assay) and expressed as AU (invasion)/ AU (MTT).

### Tumor development on chick chorioallantoic membrane (CAM)

Tumor development on CAM was performed as previously described [29]. Briefly, fertilized eggs (animal facility of the University of Geneva, Geneva, Switzerland) were incubated at 38 °C with 80% relative humidity and periodic rotation. On egg development day (EDD) 4, the eggs were drilled at their narrow apex, and the hole was closed with adhesive tape. The eggs were then incubated at 38 °C with 80% relative humidity without rotation. On EDD8 the hole in the eggshell was enlarged to allow access to the CAM. After gently scratching the membrane with a needle tip, SKOV3-Red, SKOV3-Green, or SKOV3-M cells suspension (2 × 10^6^ cells in 30 μL of geltrex, Thermo Scientific) was inoculated, and the hole was hermetically covered with Parafilm®. Eggs were returned to the incubator to allow tumor growth. Tumor growth was then monitored at EDD10.5 and 13.5 using a Wild Heerbrugg M3Z microscope at 10x magnification with a Lumenera INFINITY2-1 CDD camera with Infinity Capture Software.

### Microarray

Microarray-based transcriptome profiling was performed at the iGE3 Genomics core-facility of the faculty of medicine of the University of Geneva. Briefly, 100 ng of total RNA were extracted from SKOV3-Red, SKOV3-Green, and SKOV3-M at two different passages and were used as input for the preparation of single-strand cDNA using the WT PLUS reagent kit from Thermofisher Scientific. Targets were then fragmented and labeled with the Affymetrix GeneChip WT Terminal Labeling Kit and hybridized on Human Clariom S arrays according to the manufacturer’s recommendations.

The data were analyzed with the Transcription Analysis Console (TAC) software (ThermoFisher Scientific), selecting the genes which expression was modified at least 2-fold with a *p*-value < 0.001.

### Statistical analysis and reproducibility

Data were represented as means ± SEM. Statistical differences between samples were assessed by the Student’s t-test. A *p*-value < 0.05 was considered significant. GraphPrism software was used to perform the different statistical analyses. The number of repeated experiments was described in each method description.

## Results

### ER stress enhances PGCCs formation

**Fig. 1:**
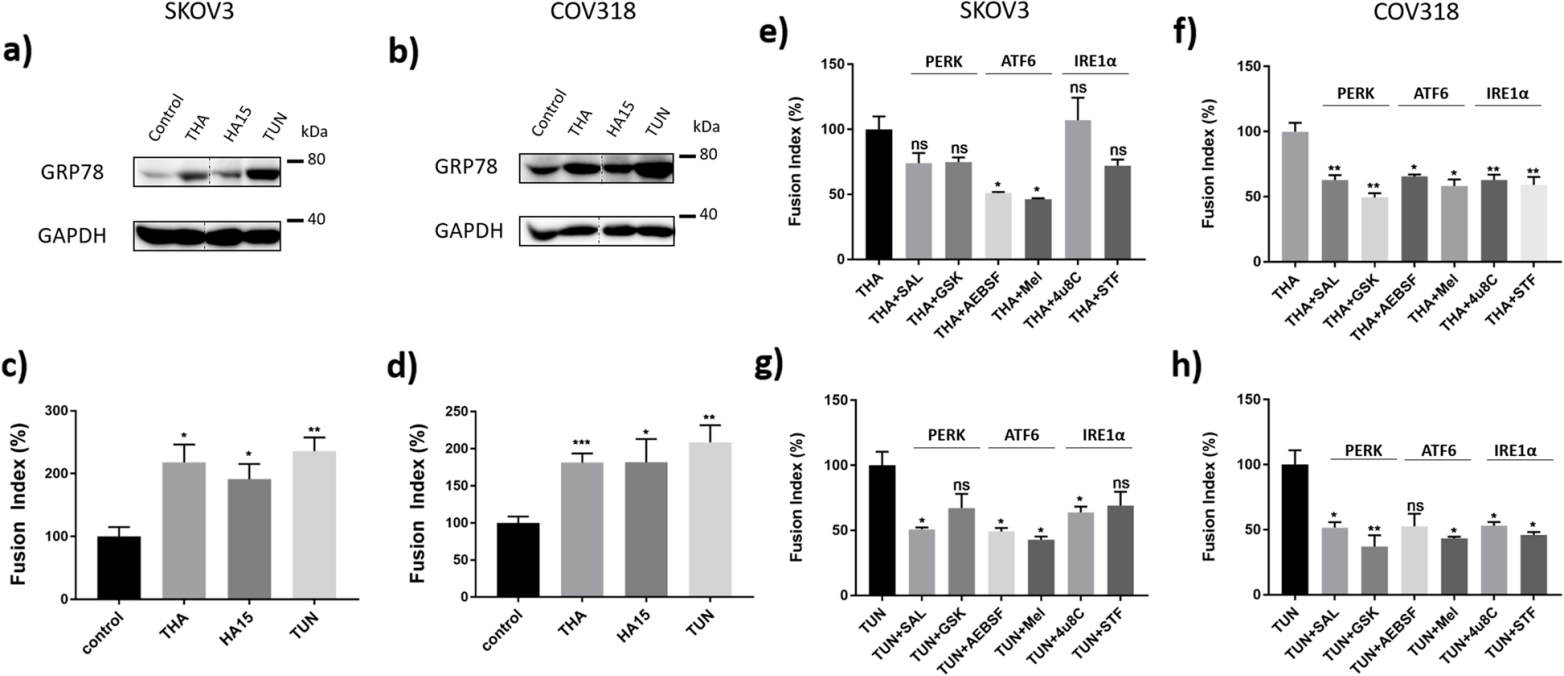
Endoplasmic reticulum (ER) stress induction enhances cell fusion in ovarian cancer cells. **a **to** d** SKOV3 (**a **and** c**) and COV318 (**b **and** d**) cells were treated with three different ER stress inducers, 100 nM Thapsigargin (THA), 1 μM HA15 or 1 μg/mL Tunicamycin (TUN) for 48 h. Untreated cells were used as the control condition. **a **and** b** Protein levels for GRP78 and GAPDH were assessed by Western blotting. The representative images of bands for GRP78 and GAPDH were taken from the same gel (Supplementary Fig. 2), and each image was cropped, as delineated in Supplementary Fig. 2, as well as adjusted for image intensity for optimal visualization. **c **and** d** Fusion index was calculated for the different treatments and expressed as percentages relative to the control condition. Results are expressed as mean ± SEM. * *p* < 0.05, ** *p* < 0.01, *** *p* < 0.001. **e **to** h** SKOV3 (**e**,** g**) and COV318 (**f**,** h**) cells were treated with either 100 nM Thapsigargin (THA) (**e**,** f**) or 1 μg/mL Tunicamycin (TUN) (**g**,** h**), combined with inhibitors of the three UPR pathways: 17.5 μM Salubrinal (SAL) or 0.3 μM GSK2656157 (GSK) for PERK pathway inhibition, 200 μM 4-(2-aminoethyl) benzenesulfonyl fluoride hydrochloride (AEBSF) or 1 mM Melatonin (Mel) for ATF6 pathway inhibition, and 8 μM 4μ8C or 2 μM STF-083010 (STF) for IRE1α pathway inhibition, for 48 h. Fusion index was expressed as percentages relative to the fusion index calculated for either the THA or TUN condition. Results are expressed as mean ± SEM. ns: non-significant, * *p* < 0.05, ** *p* < 0.01

Several studies showed that PGCCs formation in ovarian cancer cells is enhanced by hypoxic conditions, achieved either with CoCl_2_ treatment or with 0.1% oxygen cell culture [3, 13–15]. Hypoxic conditions are characteristics of the tumor environment and are known to activate UPR [30]. We previously described how ovarian cancer cells purified from malignant ascites were spontaneously able to form PGCCs in vitro [11], under regular culture conditions and that the UPR is involved in trophoblastic and choriocarcinoma cell fusion [31]. Therefore, we hypothesized that UPR could be involved in ovarian PGCC formation. To test this hypothesis, SKOV3 and COV318 cells were treated for 48 hours with three different ER stress inducers: THA, TUN, and HA15. Treatment with each of these drugs resulted in an increased protein level of GRP78, in both COV318 and SKOV3 cell lines (Fig. 1a, b), testifying of UPR activation. UPR activation was associated with a significant increase (about a 2-fold increase) in PGCC proportion, in all the tested conditions (Fig. 1c, d).

To confirm the potent role of UPR in PGCC formation, we then used molecules known to inhibit the signaling from downstream effectors of IRE1, PERK, and ATF6. Since no specific inhibitor of the ATF6 pathway was available, we used AEBSF (which blocks S1P and S2P in the Golgi apparatus, preventing ATF6 cleavage [32]) and Mel [33] to block the ATF6 pathway. STF and 4μ8C were used to inhibit the IRE1α Rnase activity [34, 35]. Finally, GSK (an ATP competitive inhibitor [36]) and SAL (an inhibitor of EIF2α dephosphorylation [37]) were used to modulate PERK pathway activation. Simultaneous treatment of SKOV3 cells with THA and each of the UPR inhibitors individually resulted in a general tendency of lowered fusion rates when compared to cells treated with THA alone (Fig. 1e). However, the only significant reduction was achieved when treating the cells with ATF6 pathway inhibitors. In contrast, treatment of SKOV3 cells simultaneously with TUN and each of the UPR inhibitors individually showed a more stringent reduction in cell fusion rate, significant for all of the inhibitors, except GSK and STF (Fig. 1g). In the same way, COV318 treatment with THA or TUN and the different UPR inhibitors resulted in a significant cell fusion rate decrease, except for the combination of TUN + AEBSF (Fig. 1f, h). Together, these results confirm the role of the UPR in the induction of ovarian cancer cell fusion.

### Cell fusion is involved in PGCCs formation

**Fig. 2:**
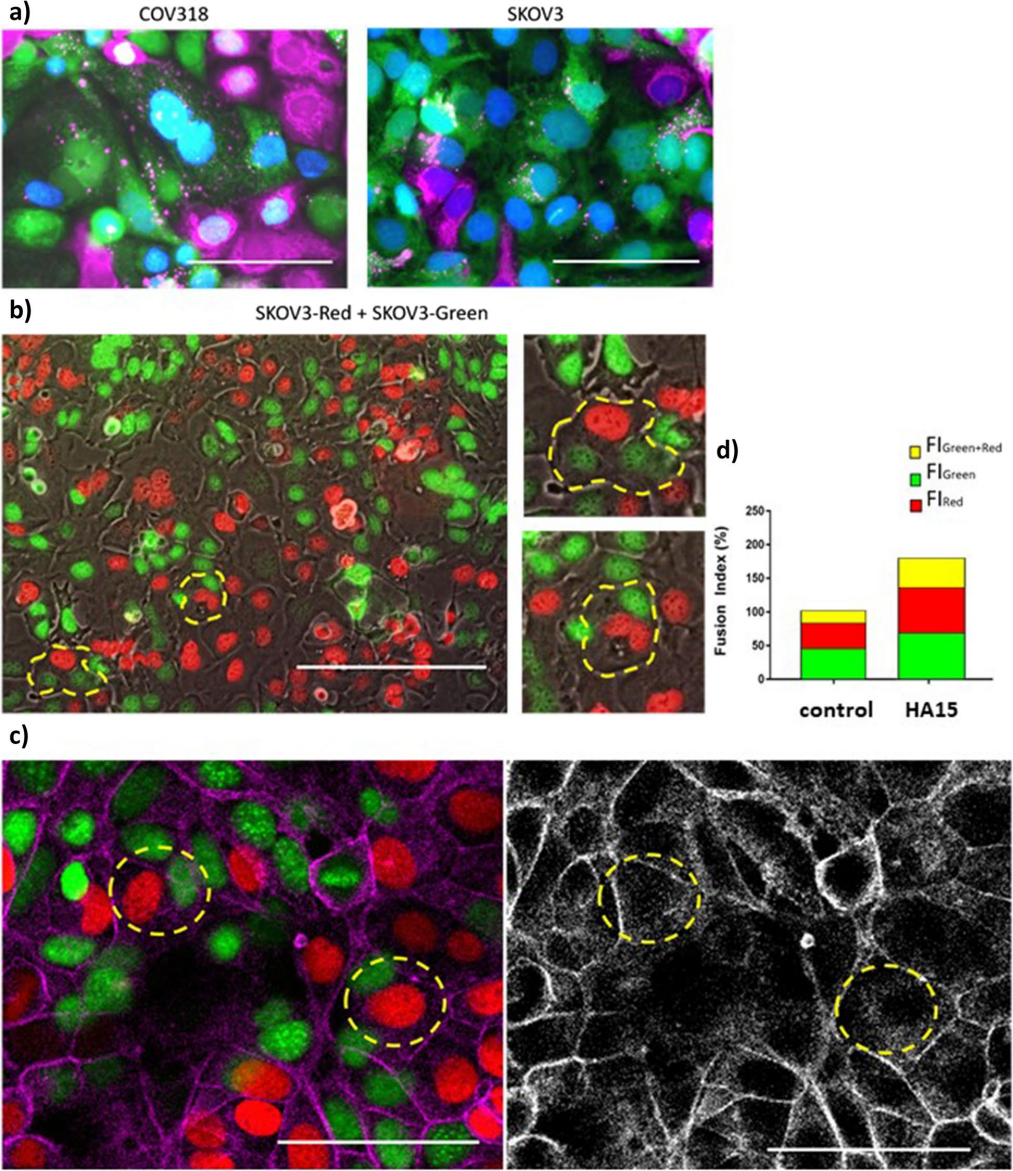
Formation of ovarian cancer PGCCs by cell fusion. **a** Ovarian cancer cells (COV318 and SKOV3) double-positive for green and far-red cytoplasmic dyes were sorted by flow cytometry following the induction of cell fusion with 300 μM CoCl_2_ for 48 h. Scale bars represent 100 μm. **b** SKOV3 cells were transfected with plasmids encoding for red (SKOV3-Red) and green (SKOV3-Green) nuclear fluorescent proteins and mixed in co-culture. Under regular culture conditions, cells spontaneously formed PGCCs by cell fusion, visualized by polynucleated cells with both red and green nuclei (circled in yellow). Scale bars represent 200 μm (with two high magnifications of selected portions at the bottom). **c **and** d** Co-culture of SKOV3-Green and SKOV3-Red cells was treated with 1 μM HA15 for 48 h. **c** The cells were labeled with Phalloidin (actin, pink/grey). The presence of polynucleated cells with both red and green nuclei was detected by confocal microscopy. Scale bars represent 100 μm. **d** Fusion index was calculated separately for green polynucleated cells (Fl_Green_), red polynucleated cells (Fl_Red_), and red/green polynucleated cells (Fl_Green + Red_). Results are expressed as mean percentages relative to the fusion index calculated for the control condition

To determine whether PGCCs originate either from endoreplication and incomplete cell division or from cell fusion, we first used the strategy described by Uygur et al. [38] in their study of cell fusion between prostate cancer and muscle cells. Ovarian cancer cell lines, SKOV3 and COV318, were stained with two different fluorescent cytoplasmic markers. The two stained cell populations were then mixed and grown in co-culture. Cells positive for both cytoplasmic markers, which we initially considered as fused cells, were sorted by flow cytometry and seeded in culture dishes. Unexpectedly, the outcome of the cell sorting was not formed mostly by fused cells that mixed their cytoplasm; instead, it was constituted by a mix of polynucleated cells and mononucleated cells that may have exchanged vesicles with cells stained with the opposite cytoplasmic marker (Fig. 2a). This result is consistent with the one published by Miroshnychenko et al. [39] and evidenced that this approach is not valid to isolate ovarian fused cells since vesicle exchange happened.

We then decided to transfect SKOV3 cells with plasmids encoding for a nuclear protein tagged with red or green fluorescent proteins, combined with two distinct antibiotic resistance genes. Following the antibiotic selection of transfected cells, we obtained homogenously nuclei-marked cell lines that we named SKOV3-Red and SKOV3-Green cell lines. After mixing and growing the two populations of cells, we observed spontaneous formation of three types of PGCCs: PGCCs with only red nuclei, PGCCs with only green nuclei, and PGCCs with both red and green nuclei (Fig. 2b). Although the two first types of PGCCs (with single-color nuclei) do not allow determining whether these cells originate from endoreplication or from cell fusion, the third type of PGCCs that contain both red and green nuclei demonstrate that the cell fusion process is involved in the generation of these PGCCs.

To determine if the UPR-mediated PGCC formation involves a cell fusion process, we induced ER stress in a co-culture of SKOV3-Red and SKOV3-Green cells with HA15 and then counted the number of PGCCs with only red nuclei, only green nuclei, and both red and green nuclei, using confocal microscopy. The proportion of PGCCs increased more than 1.3 fold in single-colored PGCCs (Red PGCCs: 1.58% ± 0.52 (SD) in CTL vs 2.11% ± 0.89 (SD) in HA15; Green PGCCs: 1.61% ± 0.52 (SD) in CTL vs 2.23% ± 0.68 (SD) in HA15), and more than 2.1 fold in PGCCs with both red and green nuclei (Red + Green PGCCs: 0.74% ± 0.34 (SD) in CTL vs 1.57% ± 0.71 (SD) in HA15). The calculation of the fusion index, which considers the number of nuclei contained within the syncytia, showed that HA15-induced ER stress enhanced the formation of each of the three types of PGCCs, including Red + Green PGCCs, formed by cell fusion (Fig. 2c, d). These results confirm that cell fusion is involved in the formation of PGCCs and that UPR could increase this process in ovarian cancer cells.

**Fig. 3:**
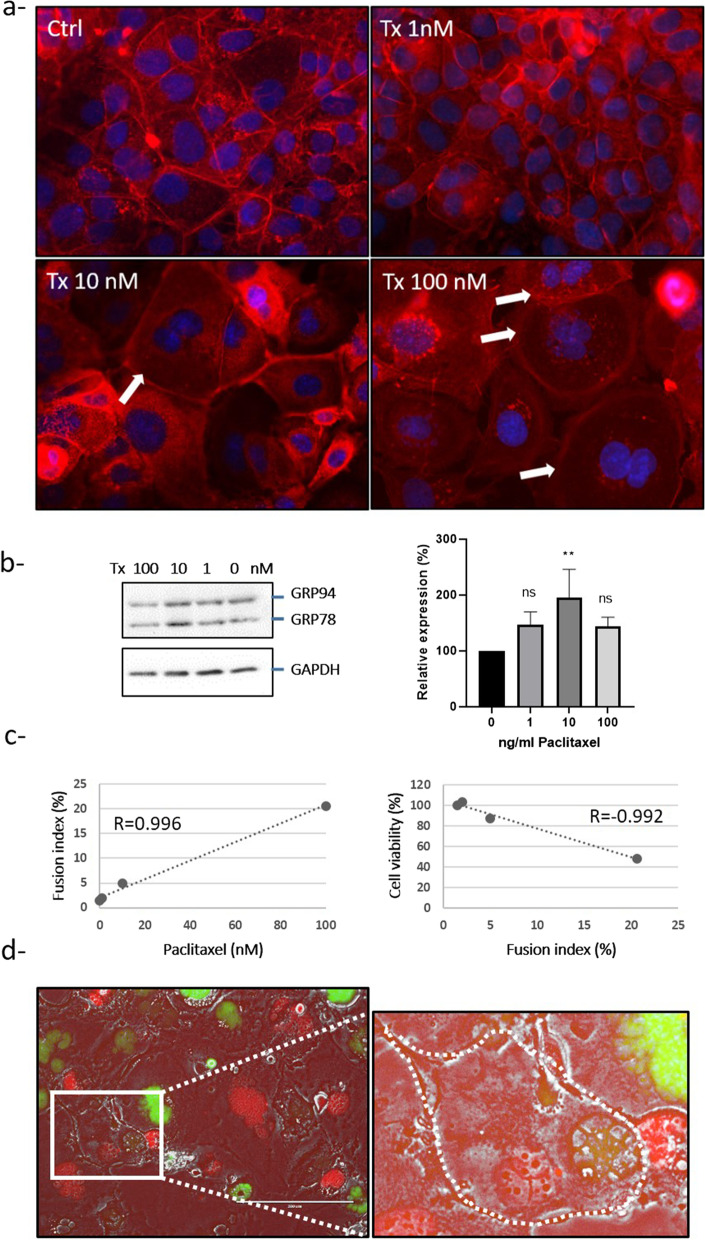
Paclitaxel treatment induces ovarian PGCC formation. **a**,** b **and** c** SKOV3 cells were treated with 0, 1, 10 and 100 nM paclitaxel (Tx) for 48 h. **a** The cells were labeled with DAPI (nucleus, blue) and Phalloidin (actin, red) and observed by fluorescence microscopy at the original magnification of 40×. The white arrow indicates the multinucleated cell. **b** Protein levels for GRP78 and GAPDH were assessed by Western blotting. GRP78 and GAPDH (used as loading control) were quantified using the ImageJ software, and data are expressed as the GRP78/GAPDH fold change relative to the control. **c** Fusion index and cell viability (MTT assay) were evaluated. A correlation between fusion index and paclitaxel treatment or cell viability was then calculated. R: Pearson correlation. **d** Co-culture of SKOV3-Green and SKOV3-Red cells was treated with 10 nM Paclitaxel for 48 h. **c** The presence of polynucleated cells with both red and green nuclei was detected by fluorescence microscopy (delimited with a white dashed line). The picture represents the overlay of red signal (mCherry), green signal (GFP) and phase contrast. The white boxed area is shown at a higher magnification to the right and (X3) and brightness. Scale bar, 200 μm; original magnification, × 20

As chemotherapeutic treatment is known to induce the UPR [40], we then treated SKOV3 cells with different concentrations of paclitaxel. We confirmed that this treatment induced UPR activation, attested by the increase in GRP78 expression in SKOV3 cells (Fig. 3b) and PGCC formation (Fig. 3a and c). We also observed a correlation between fusion index (representative of PGCC) and paclitaxel concentration or cell death (Fig. 3c).

To determine if paclitaxel-induced PGCC formation could be due, at least in part, to cell fusion, we next incubated both SKOV3-Green and SKOV3-Red cells with 10 nM paclitaxel for 48 h before changing the culture medium. Most of the polyploid cells observed 6 days after paclitaxel treatment had only green or red nuclei and may be mainly due to mitosis failure and endoreplication as described by Niu et al. [13]. However, some cells contained both red and green nuclei suggesting that these cells were also able to fuse under paclitaxel treatment (Fig. 3d).

### PGCCs can revert to mononucleated cells under selection pressure and reinitiates normal cell cycles

**Fig. 4:**
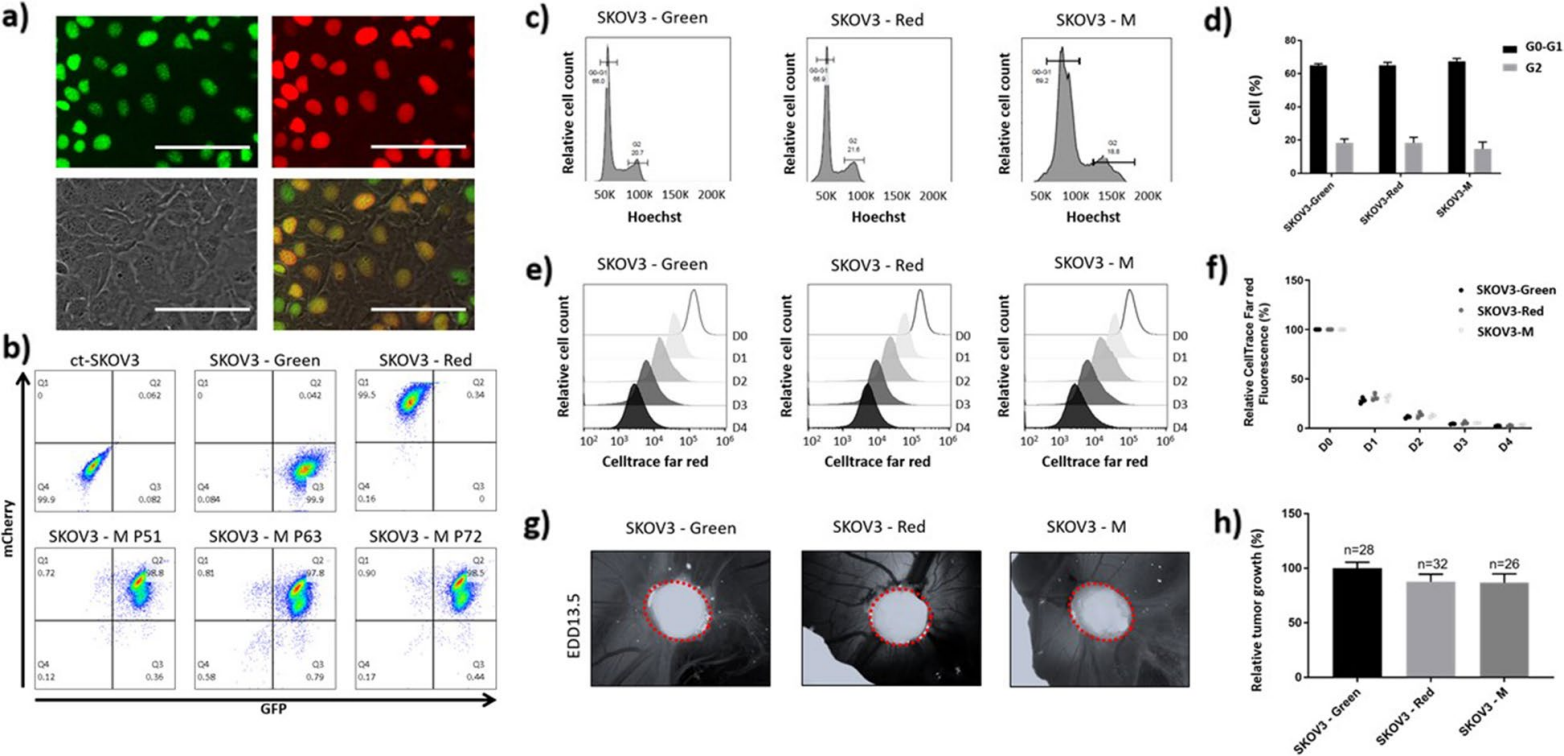
Ovarian cancer PGCCs can proliferate and form tumors on chick chorioallantoic membrane (CAM). SKOV3-Red and SKOV3-Green were mixed in co-culture with double-antibiotic. **a **and** b** Cell population (SKOV3-M) obtained after 3 weeks of double selection. **a** Visualization of double-positive mononucleated cells by fluorescent microscopy. Upper left: green signal (GFP). Upper right: red signal (mCherry). Lower left: Phase contrast. Lower right: Overlay. Scale bars represent 100 μm. **b** Quantification of red (mCherry), green (GFP), and double-positive cells by flow cytometry in SKOV3-Red, SKOV3-Green, SKOV3-M, and untransfected SKOV3 cells (ct-SKOV3). SKOV3-M cells were analyzed at passages 51, 63, and 72. **c **and** d** Cell cycle distribution was analyzed in SKOV3-Red, SKOV3-Green, and SKOV3-M cells by flow cytometry following Hoechst staining. Doublets cells have been removed from the analysis based on Hoescht W and Hoescht A signals. **c** Cell distribution depending on Hoechst signal intensity. **d** Percentages of cells in G0-G1 and G2 phases. **e **and** f** Proliferation capacities of SKOV3-Red, SKOV3-Green, and SKOV3-M cells were assessed by analyzing CellTrace™ far-red cytoplasmic signal dilution by flow cytometry. Cells were stained on day 0 (D0) and seeded in 10 cm culture dishes (5 × 10^5^ cells/dish). Far-red signal was analyzed for five consecutive days (D0 to D4). Doublets cells have been removed from the analysis based on SSC W and SSC A signals. **e** Evolution of far-red signal intensity from D0 to D4. **f** Evolution of far-red signal dilution expressed as a percentage of mean signal intensity ± SEM measured at D0. **g **and** h** Tumorigenic capacity of SKOV3-Red, SKOV3-Green, and SKOV3-M cells was assessed using the CAM model. Cells were inoculated on egg development day (EDD) 8, and tumor size was measured at EDD10.5 and EDD13.5. **g** Images of the tumors growing in CAM at EDD13.5 **h** Tumor growth (tumor size at EDD13.5 relative to tumor size at EDD10.5). Results are expressed as mean ± SEM

To investigate the fate of ovarian PGCCs formed by cell fusion, we selected red and green fluorescent SKOV3 cells derived from co-culture of SKOV3-Red with SKOV3-Green under double antibiotic selection, which we named SKOV3-M. Indeed, fused cells containing both types of nuclei would be able to survive a double antibiotic selection, while the unfused parental cell lines and the same type of nuclei-fused cells would die under this condition. Strikingly, after 3 weeks under double antibiotic selection, we observed that SKOV3-M cells were no longer polynucleated and that their single nucleus was positive for both fluorescent proteins (Fig. 4a), suggesting that the mixing of genetic material had occurred. Flow cytometry analysis confirmed that about 98% of SKOV3-M cells were positive for both red and green fluorescent proteins, and this phenotype was maintained over passage 72 (Fig. 4b).

Since SKOV3-M appeared to have restarted a normal cell growth, we determined if they were able to achieve normal cell cycles. We observed a similar G0-G1 and G2 phase cell distribution between SKOV3-M and its parental cell lines, with about 66 to 70% of cells in the G0-G1 phase, and about 18 to 22% of cells in the G2 phase (Fig. 4c, d). Interestingly, the Hoechst staining was more intense in SKOV3-M cells (Fig. 4c), suggesting a higher DNA content in the fused cells than in the parental ones. Additionally, we decided to study the proliferative capacities of SKOV3-M cells by staining the cells with CellTrace™ far-red. The division profile of stained cells was analyzed during five consecutive days. SKOV3-M and its parental cell lines had proliferated and diluted CellTrace™ far-red fluorescence in the same way (Fig. 4e, f).

We then compared the tumor growth of SKOV3-M and its parental cells-derived tumors using a chick chorioallantoic membrane (CAM) model. The tumor size (Fig. 4g) and tumor growth (Fig. 4h) were not significantly different between SKOV3-M and the parental cell lines.

### Comparison of SKOV3-M omics profile with its parental cell lines

**Fig. 5:**
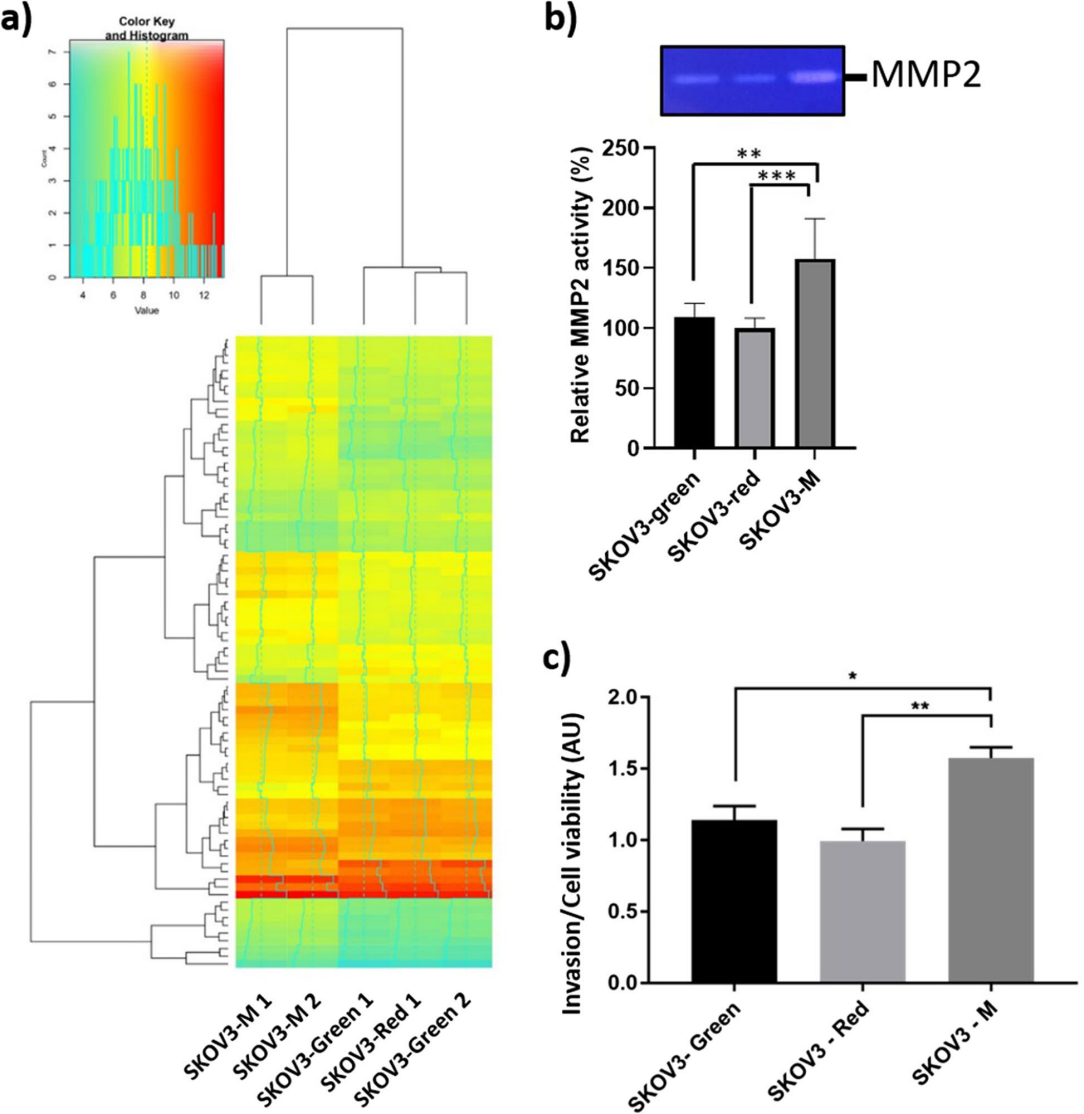
SKOV3-M cells display differential gene expression and invasive capacity than its parental cell lines. **a** Heat maps of genes expression in SKOV3-M, SKOV3-Green, and SKOV3-Red. Colors from yellow to blue indicate downregulated cellular genes; colors from yellow to red indicate upregulated cellular genes. **b** MMP-2 activity in the culture supernatant of SKOV3-M, SKOV3-Green, and SKOV3-Red. White bands were quantified using the ImageJ software, and data were normalized by protein concentration and expressed as the fold change relative to the control. The original gel of the three experiments is shown in supplementary Fig. 5. **c** The invasive capacity of SKOV3-Red, SKOV3-Green, and SKOV3-M cells was assessed by the Boyden chamber assay. Cell viability was assessed by MTT assay. Results are expressed as invasion relative to cell viability ± SEM. AU: arbitrary unit, * *p* < 0.05, ** *p* < 0.01, ***p < 0.001

Higher DNA content found in SKOV3-M cells may be associated with an important chromosomal rearrangement, which is known to alter the regulation of gene expression [41] and exacerbate tumor malignancy [42]. We thus decided to compare the transcriptome of SKOV3-M to the parental cell lines. Comparative transcriptomic analysis of SKOV3-M to the parental cell lines identified 82 genes that were differentially expressed (+/− 2-fold, *p* ≤ 0.001; Fig. 5a; see Supplementary Table 1 for the 10 most significantly upregulated and downregulated genes). The alteration of some proteins’ expression in SKOV3-M compared to the parental cell lines was confirmed by secretomic analysis (Supplementary Fig. 1, Supplementary Table 2, Supplementary Table 3). Among differentially expressed genes, enrichment of biological processes identified cell adhesion, locomotion, and invasion to be among the statistically altered processes (Table 1). In addition, according to TAC analysis, the 4 signaling pathways which may be altered in SKOV3-M with the highest significance are the malignant plural mesothelioma (*p* = 0.009), epithelial to mesenchymal transition in colorectal cancer (*p* = 0.035), cell migration and invasion through p75NTR (*p* = 0.07) and AXL signaling pathways (*p* = 0.06). MMP2, which is known to be involved in cell migration and invasion, is the common upregulated gene in these 4 pathways. We thus evaluated MMP2 activity in culture supernatant of SKOV3-M and its parental cell lines. We confirmed an increased MMP2 activity in culture supernatant of SKOV3-M compared to its parental cell lines (Fig. 5b). Therefore, we hypothesized that ovarian cancer cell fusion confers enhanced invasive capacities to SKOV3-M cells. To test this hypothesis, the three cell lines SKOV3-Green, SKOV3-Red and SKOV3-M were seeded in Boyden chamber. The capacity of SKOV3-M to invade collagen was increased compared to its parental cell lines (Fig. 5c), suggesting that cell fusion can modify the properties of ovarian cancer cells.

**Table 1:** Cell migration, cell motility, invasion, cell adhesion and locomotion-related genes, the expression of which is at least 2-fold modified and *p*-value of which is < 0.05 in SKOV3-M cells compared to SKOV3-Green and SKOV3-Red cells

Gene symbol	Fold change	***P***-value	Function
IL1R2	11.03	5.98E-06	Cell migration
MMP2	8.13	0.002	Invasion
MXRA5	7.09	2.79E-05	Invasion
VEGFC	3.57	0.0108	Invasion
AFAP1L2	3.1	0.0012	Cell motility
EFNB2	2.71	2.95E-05	Invasion
LRRC17	2.5	0.0032	Locomotion
ADGRE5	2.49	1.96E-05	Cell adhesion
ELFN2	2.34	0.0002	Cell migration
FBLN5	−2.02	0.0043	Cell adhesion
BCAM	−2.15	0.0012	Cell adhesion
CDHR1	−2.54	6.17E-05	Cell adhesion
ITGB4	−2.56	0.0197	Cell adhesion
IL7R	−3	0.0064	Invasion
ADAMTSL4	−3.33	0.0042	Cell adhesion
EPCAM	−3.99	1.31E-06	Cell adhesion
IGFBP3	−5.18	0.0007	Cell migration
S1PR1	−6.54	1.65E-07	Cell migration

## Discussion

PGCC formation has been studied in several cancer types, including colon, breast, and ovarian cancer [9, 13–15]. This process is induced by DNA-damaging agents and could be linked to chemotherapeutic resistance acquisition [9, 13, 14, 43]. It could also be incremented by chemical hypoxia treatment (CoCl_2_) or hypoxic culture conditions in vitro [9, 13–15]. The mechanism for PGCC formation has always been controversial since it could be explained either by a defect in cytokinesis leading to endoreplication, by cell fusion events, or by a combination of both mechanisms [8, 9, 12]. Under chemical hypoxia, it was shown that at least 10 to 20% of ovarian PGCCs were formed by cell fusion [9]. The characterization of the PGCCs formed under these conditions displayed a modification in their division process, changing from mitosis to bursting and budding [9]. The PGCCs generated daughter cells via asymmetric division, which were able to form spheroids and expressed several markers of cancer stem cells [9]. Moreover, they were more tumorigenic than regular-sized cancer cells in the nude mice [9].

The increment in ovarian PGCC formation achieved under hypoxia in vitro pointed towards hypoxia as a possible cell fusion enhancer [9, 13–15]. Paclitaxel treatment, an antimitotic drug, was also shown to cause an induced prominence of PGCCs [[13] and Fig. 3]. A shared pathway downstream of low oxygen tension and paclitaxel treatment is the UPR pathway ([17, 19, 44–50], Fig. 3). We thus hypothesized that UPR activation may result in cancer cell fusion enhancement and PGCC formation. The activation of the UPR with different UPR activators effectively increased cell fusion and PGCCs formation with both SKOV3 and COV318 cell lines. On the contrary, the inhibition of the UPR pathways by inhibitors targeting the three UPR-branches led to a general tendency for decreased cell fusion, as already observed in choriocarcinoma cell line [24, 25]. Therefore, we can conclude that the UPR, through the activation of the UPR sensors ATF6, IRE1α, and PERK, is involved in ovarian PGCC formation. However, the contribution of each UPR pathway and their interdependence are yet to be elucidated.

**Fig. 6:**
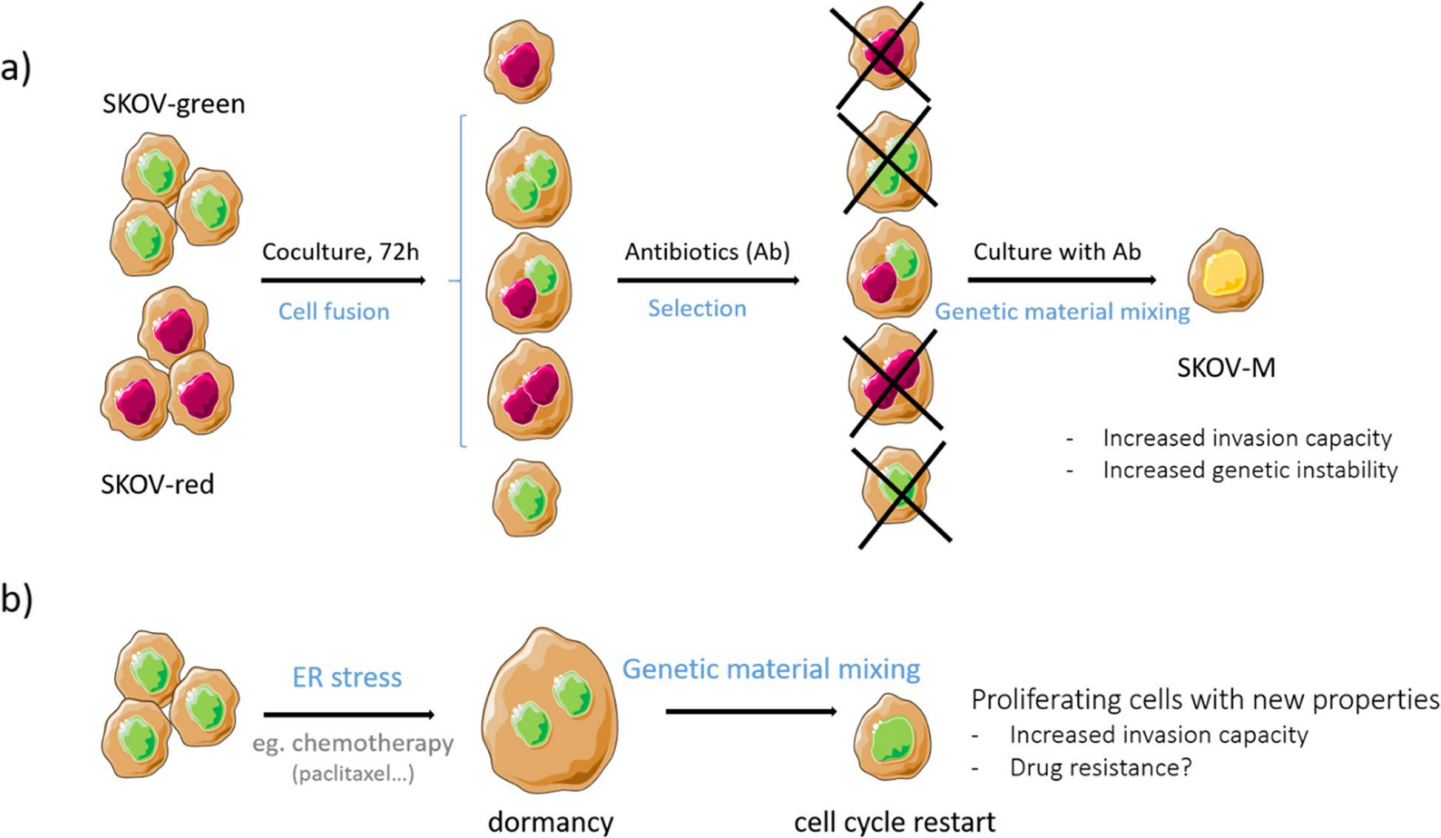
Following the experimental procedure we used to obtain SKOV-M (**a**), we hypothesized that some cancer cells exposed to ER stress such as during chemotherapy treatment, fuse to form PGCCs. These polyploid cells are in dormancy and are consequently, unaffected by drugs targeting dividing cells. After genetic material mixing, these cells are able to proliferate and acquire new properties such as invasiveness and possibly drug resistance, associated with malignancy (**b**)

Despite PGCCs could generate a more aggressive daughter, the mechanism and conditions initiating their formation have been poorly studied. In the present study, using plasmids expressing nuclear proteins tagged with green or red fluorochrome, we generated PGCCs from SKOV3 cells and confirmed that these PGCCs were at least partially derived from cell fusion events. In addition, we described for the first time, that under double antibiotic selection pressure, mononucleated cells containing both red- and green-fluorescent nuclear proteins, can be selected, suggesting that mixing of genetic material had occurred. Despite higher DNA content, SKOV3-M cells resumed a normal cell cycle and were able to proliferate in the same manner as their parental cell lines (SKOV3-Green and SKOV3-Red). This novel observation leads us to hypothesize that genetic material mixing may be a mechanism by which PGCCs, after surviving drug treatment, could return the cell cycle to mitosis mitosis (Fig. 6a).

This mechanism could be transposed to ovarian cancer cells exposed to chemotherapy (Fig. 6b). ER stress induced by chemotherapy enhances PGCC formation. PGCCs are in dormancy and are no longer affected by drugs targeting dividing cells. When conditions become propitious, these cells may mix their genetic material and proliferate again.

Transcriptomic analysis of the SKOV3-M cells showed that more than 80 genes were at least 2-fold modulated in comparison with their parental cells. Among them, we found genes implicated in cell adhesion, motility, and invasion. The validation of these results with invasion assays demonstrated the increased invasive properties of SKOV3-M cells compared to their parental cells. This increased invasiveness renders the cells more aggressive, favoring the onset of metastases [51–53]. Interestingly, our results are supported by a recent study, combining in vitro, in vivo, and in silico experimental models, in which the authors suggest that the genetic recombination, subsequent to cancer cell fusion they observed in vitro and in vivo, can facilitate cancer cell adaptation and therefore tumor progression [39].

The proliferation rate of these fusion-derived cells, their capacities to resist both antibiotics, to form tumors, which we measured *in ovo* on a CAM model and their invasive properties may explain the high cancer relapse rates that ovarian cancer patients suffer. However, the question of whether ovarian cancer cell fusion occurs in vivo remains.

## Conclusions

A growing body of evidence has shown that the UPR is involved in drug resistance in different cancer cells [46–48, 50]. However, the implication of the UPR in chemotherapy resistance is complex and not fully understood. In this study, we showed that paclitaxel treatment induces ovarian PGCC formation in vitro. Most of these PGCC could be due to mitosis failure and endoreplication, but some of them were formed by cell fusion. We demonstrated that decreasing the UPR activation in ovarian cancer cells could decrease ovarian cancer cell fusion and PGCC formation. Avoiding PGCC formation may reduce cancer relapses and metastasis, while at the same time, the drug resistances generated by the UPR could be prevented [9, 46, 47, 49]. A better understanding of the molecular mechanism controlling UPR-mediated PGCCs formation and drug resistance is highly needed to develop new combined therapeutic approaches with drugs that specifically target the UPR sensors or downstream partners and conventional chemotherapy.

## Supplementary Information

**Additional file 1: Supplementary Table 1.** The 10 most significantly upregulated (in grey) and downregulated (in white) genes in SKOV3-M compared to its parental cell lines. **Supplementary Table 2.** Proteins only found in secretome of SKOV3-M. **Supplementary Table 3.** Proteins only found in secretome of SKOV3-red and SKOV3-green. **Supplementary Figure 1.** Protein Venn diagram. Culture supernatants of SKOV3-M (*n* = 4), SKOV3-Green (*n* = 2) and SKOV3-Red (*n* = 2) were analyzed by liquid chromatography-electrospray ionization-mass spectrometry/mass spectrometry. The number of identified proteins is indicated for each cell line. **Supplementary Figure 2.** Full images of western blots shown in Fig. 1. A- SKOV3 cells treated with UPR modulators. B- COV318 treated with UPR modulators. Red dotted lines indicate the cropping locations. THA: thapsigargin; TUN: tunicamycin; SAL: salubrinal; GSK: GSK2656157; STF: STF-083010. **Supplementary Figure 4.** Full images of western blots shown in Fig. 3. SKOV3 cells treated with Paclitaxel (0, 1, 10 and 100 nM). A- Red dotted lines indicate the cropping locations. Ptx: Paclitaxel; n1: first experiment; n2: second experiment. B- Pictures of membranes C- Original images at different exposition time. Red dotted lines indicate the cropping locations used for images shown in A-. **Supplementary Figure 5.** Full image of zymogram shown in Fig. 5. SKOV3-Green (lines 2, 5 and 9), SKOV3-Red (lines 3, 6, and 10), SKOV3-M (lines 4, 7 and 11) and conditioned medium of MCF7 (line 1, marker). Red dotted lines indicate the cropping locations. N1: first experiment; n2: second experiment; n3: third experiment.

## Data Availability

The datasets generated during and/or analyzed during the current study are available from the corresponding author on reasonable request.

## Abbreviations

AEBSF: 4-(2-aminoethyl) benzenesulfonyl fluoride hydrochloride;
ATF6: Activating transcription factor 6
CAM: Chick chorioallantoic membrane
CoCl_2_: Cobalt chloride
CSC: Cancer stem cell
EDD: Egg development day
EOC: Epithelial ovarian cancer
ER: Endoplasmic reticulum
GRP78: Glucose-regulated protein 78
GSK: GSK2656157
IRE1α: Inositol-requiring enzyme 1α
Mel: Melatonin
PERK: Protein kinase RNA-like endoplasmic reticulum kinase
PGCC: Polyploid giant cancer cell
SAL: Salubrinal
STF: STF-083010
TAC: Transcription analysis console
THA: Thapsigargin
TUN: Tunicamycin
UPR: Unfolded protein response
