# Supplementary material for: Linking unfolded protein response to ovarian cancer cell fusion
Source: BMC Cancer. 2022 Jun 7;22:622. doi: 10.1186/s12885-022-09648-4 (PMC9172076; doi:10.1186/s12885-022-09648-4)
Supplement: Supplementary file 1 — Additional file 1: Supplementary Table 1. The 10 most significantly upregulated (in grey) and downregulated (in white) genes in SKOV3-M compared to its parental cell lines. Supplementary Table 2. Proteins only found in secretome of SKOV3-M. Supplementary Table 3. Proteins only found in secretome of SKOV3-red and SKOV3-green. Supplementary Figure 1. Protein Venn diagram. Culture supernatants of SKOV3-M (n = 4), SKOV3-Green (n = 2) and SKOV3-Red (n = 2) were analyzed by liquid chromatography-electrospray ionization-mass spectrometry/mass spectrometry. The number of identified proteins is indicated for each cell line. Supplementary Figure 2. Full images of western blots shown in Fig. 1. A- SKOV3 cells treated with UPR modulators. B- COV318 treated with UPR modulators. Red dotted lines indicate the cropping locations. THA: thapsigargin; TUN: tunicamycin; SAL: salubrinal; GSK: GSK2656157; STF: STF-083010. Supplementary Figure 4. Full images of western blots shown in Fig. 3. SKOV3 cells treated with Paclitaxel (0, 1, 10 and 100 nM). A- Red dotted lines indicate the cropping locations. Ptx: Paclitaxel; n1: first experiment; n2: second experiment. B- Pictures of membranes C- Original images at different exposition time. Red dotted lines indicate the cropping locations used for images shown in A-. Supplementary Figure 5. Full image of zymogram shown in Fig. 5. SKOV3-Green (lines 2, 5 and 9), SKOV3-Red (lines 3, 6, and 10), SKOV3-M (lines 4, 7 and 11) and conditioned medium of MCF7 (line 1, marker). Red dotted lines indicate the cropping locations. N1: first experiment; n2: second experiment; n3: third experiment. [file 12885_2022_9648_MOESM1_ESM.docx]

**Supplementary information**

**Supplementary Methods**

**Secretomic**

Secretome profiles were analyzed and compared for SKOV3-Green, SKOV3-Red, and SKOV3-M cells at the Proteomic core-facility of the faculty of medicine of the University of Geneva. The cells were seeded in 35 mm culture dishes (6 × 10^5^ cells/dish) in a regular complete culture medium. Twenty-four hours after seeding, the culture medium was discarded and replaced by 1.5 mL/dish of culture medium without FBS. The cells were cultured in these conditions for 48h. The supernatants were then harvested, centrifuged at 11 000 RPM for 5 min at 4°C to eliminate cell fragments, and stored at -80°C until processing. Adherent cells were processed for protein extraction. The protein concentration was measured for each cell type by Bradford assay, and the whole-protein contents were used to normalize the secretomic data.

For secretomic analysis, the samples were defrosted, and the proteins were digested. The peptides were then analyzed by nanoLC-MSMS using an easynLC1000 (Thermo) coupled with a Qexactive Plus mass spectrometer (Thermo). Database search was performed with Mascot (Matrix Science) using the Human Reference Proteome. Data were analyzed and validated with Scaffold (Proteome Software) with 1% of protein FDR and at least 2 unique peptides per protein with a 0.1% of peptide FDR.

**Supplementary Tables**

**Supplementary Table 1.** The 10 most significantly upregulated (in grey) and downregulated (in white) genes in SKOV3-M compared to its parental cell lines

| **Genes symbol** | **Genes name** | **p-value** |
| --- | --- | --- |
| AKR1B15 | aldo-keto reductase family 1, member B15 | < 0.00010 |
| MXRA5 | matrix-remodelling associated 5 | < 0.0005 |
| CD274 | CD274 molecule | < 0.00010 |
| CDK15 | cyclin-dependent kinase 15 | < 0.00010 |
| GCNT2 | glucosaminyl (N-acetyl) transferase 2, I-branching enzyme | < 0.0005 |
| ENC1 | ectodermal-neural cortex 1 (with BTB domain) | < 0.0005 |
| IL1R2 | interleukin 1 receptor, type II | < 0.0005 |
| MAP2 | microtubule associated protein 2 | < 0.00010 |
| MCTP1 | multiple C2 domains, transmembrane 1 | < 0.00010 |
| AIM1 | absent in melanoma 1 | < 0.0005 |
| KRT7 | keratin 7, type II | < 0.0005 |
| MAL2 | mal, T-cell differentiation protein 2 (gene/pseudogene) | < 0.00010 |
| S1PR1 | sphingosine-1-phosphate receptor 1 | < 0.00010 |
| TOX | thymocyte selection-associated high mobility group box | < 0.0005 |
| EPCAM | epithelial cell adhesion molecule | < 0.00010 |
| RNF187 | ring finger protein 187 | < 0.00010 |
| KIT | v-kit Hardy-Zuckerman 4 feline sarcoma viral oncogene homolog | < 0.0005 |
| LURAP1L | leucine rich adaptor protein 1-like | < 0.0005 |
| DDR2 | discoidin domain receptor tyrosine kinase 2 | < 0.00010 |
| IGFBP3 | Insulin-like Growth Factor Binding Protein 3 | < 0.0005 |

**Supplementary Table 2.** Proteins only found in secretome of SKOV3-M

|  | | |  |  |
| --- | --- | --- | --- | --- |
| **PROTEIN** | **MW** | **p-value** | | |
| PSMD11 | 47 kDa | < 0.00010 | |  |
| APEH | 81 kDa | < 0.00010 | |  |
| ZNF207 | 51 kDa | < 0.00010 | |  |
| CAPN1 | 82 kDa | < 0.00010 | |  |
| CMBL | 28 kDa | < 0.00010 | |  |
| CRIP2 | 22 kDa | < 0.00010 | |  |
| DDRGK1 | 36 kDa | < 0.00010 | |  |
| DRG1 | 41 kDa | < 0.00010 | |  |
| HARS | 57 kDa | < 0.00010 | |  |
| NRP2 | 105 kDa | < 0.00010 | |  |
| NNMT | 30 kDa | < 0.00010 | |  |
| NAMPT | 56 kDa | < 0.00010 | |  |
| TPR | 267 kDa | < 0.00010 | |  |
| PAWR | 37 kDa | < 0.00010 | |  |
| S100A13 | 11 kDa | < 0.00010 | |  |
| PPP1R12A | 115 kDa | < 0.00010 | |  |
| PNPO | 30 kDa | < 0.00010 | |  |
| STK24 | 49 kDa | < 0.00010 | |  |
| SNRPE | 11 kDa | < 0.00010 | |  |
| AIFM1 | 67 kDa | < 0.00010 | |  |
| XPO1 | 123 kDa | < 0.00010 | |  |
| KTN1 | 156 kDa | < 0.00010 | |  |
| PLPBP | 30 kDa | < 0.00010 | |  |
| ARPC2 | 34 kDa | < 0.00010 | |  |
| EPB41L2 | 113 kDa | < 0.00010 | |  |
| GMPS | 77 kDa | < 0.00010 | |  |
| FAM129B | 84 kDa | < 0.00010 | |  |
| TCEA1 | 34 kDa | < 0.00010 | |  |
| PSMD8 | 40 kDa | 0.00032 | |  |
| RNPEP | 73 kDa | 0.00032 | |  |
| BZW2 | 48 kDa | 0.00032 | |  |
| PFDN2 | 17 kDa | 0.00032 | |  |
| MKI67 | 359 kDa | 0.00032 | |  |
| SNRPD3 | 14 kDa | 0.00032 | |  |
| RAD23B | 43 kDa | 0.00032 | |  |
| VPS26A | 38 kDa | 0.00032 | |  |
| ANP32A | 29 kDa | 0.00066 | |  |
| GBE1 | 80 kDa | 0.0011 | |  |
| ACTR2 | 45 kDa | 0.0011 | |  |
| QARS | 88 kDa | 0.0011 | |  |
| VPS35 | 92 kDa | 0.0011 | |  |
| PPA2 | 38 kDa | 0.0013 | |  |
| LSM2 | 11 kDa | 0.0013 | |  |
| HDGF | 27 kDa | 0.0016 | |  |
| AP2B1 | 105 kDa | 0.0032 | |  |
| EML4 | 109 kDa | 0.0032 | |  |
| SH3BGRL3 | 10 kDa | 0.0032 | |  |
| SH3BGRL | 13 kDa | 0.0032 | |  |
| ETF1 | 49 kDa | 0.0042 | |  |
| EIF3B | 92 kDa | 0.0042 | |  |
| EIF3F | 38 kDa | 0.0042 | |  |
| SF3B2 | 100 kDa | 0.0042 | |  |
| SOD2 | 25 kDa | 0.0042 | |  |
| UFD1 | 35 kDa | 0.0042 | |  |
| PGM2 | 68 kDa | 0.0059 | |  |
| ATIC | 65 kDa | 0.012 | |  |
| COPA | 138 kDa | 0.012 | |  |
| RTN4 | 130 kDa | 0.012 | |  |
| SMC1A | 143 kDa | 0.012 | |  |
| VAPA | 28 kDa | 0.012 | |  |
| CAPN2 | 80 kDa | 0.013 | |  |
| CCAR2 | 103 kDa | 0.023 | |  |
| KIF5B | 110 kDa | 0.023 | |  |
| MAP1B | 271 kDa | 0.023 | |  |
| SND1 | 102 kDa | 0.023 | |  |
| SF3B3 | 136 kDa | 0.028 | |  |
| HNRNPUL2-BSCL2 | 85 kDa | 0.033 | |  |
| IARS | 145 kDa | 0.033 | |  |
| PGLS | 28 kDa | 0.040 | |  |

**Supplementary Table 3.** Proteins only found in secretome of SKOV3-red and SKOV3-green.

| **PROTEIN** | **MW** | **p-value** |
| --- | --- | --- |
| CYR61 | 42 kDa | 0.0091 |
| CPA4 | 47 kDa | 0.024 |
| IGFBP3 | 32 kDa | 0.033 |
| FSTL3 | 28 kDa | 0.043 |

**Supplementary Figures**

**Supplementary Figure 1.** Protein Venn diagram. Culture supernatants of SKOV3-M (n= 4), SKOV3-Green (n=2) and SKOV3-Red (n= 2) were analyzed by liquid chromatography-electrospray ionization-mass spectrometry/mass spectrometry. The number of identified proteins is indicated for each cell line.


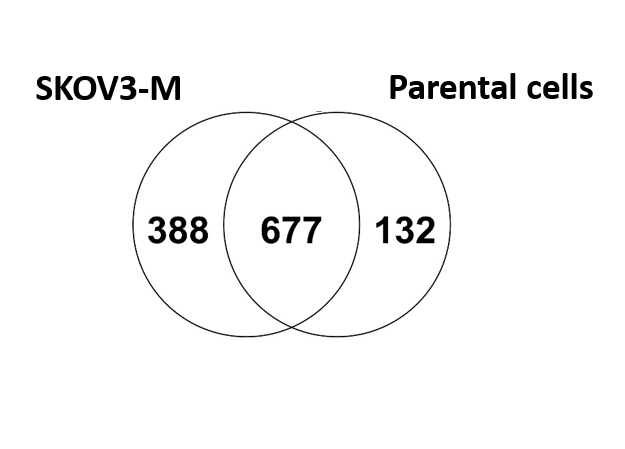


**Supplementary Figure 2.** Full images of western blots shown in Figure 1. A- SKOV3 cells treated with UPR modulators. B- COV318 treated with UPR modulators. Red dotted lines indicate the cropping locations. THA: thapsigargin; TUN: tunicamycin; SAL: salubrinal; GSK: GSK2656157; STF: STF-083010.


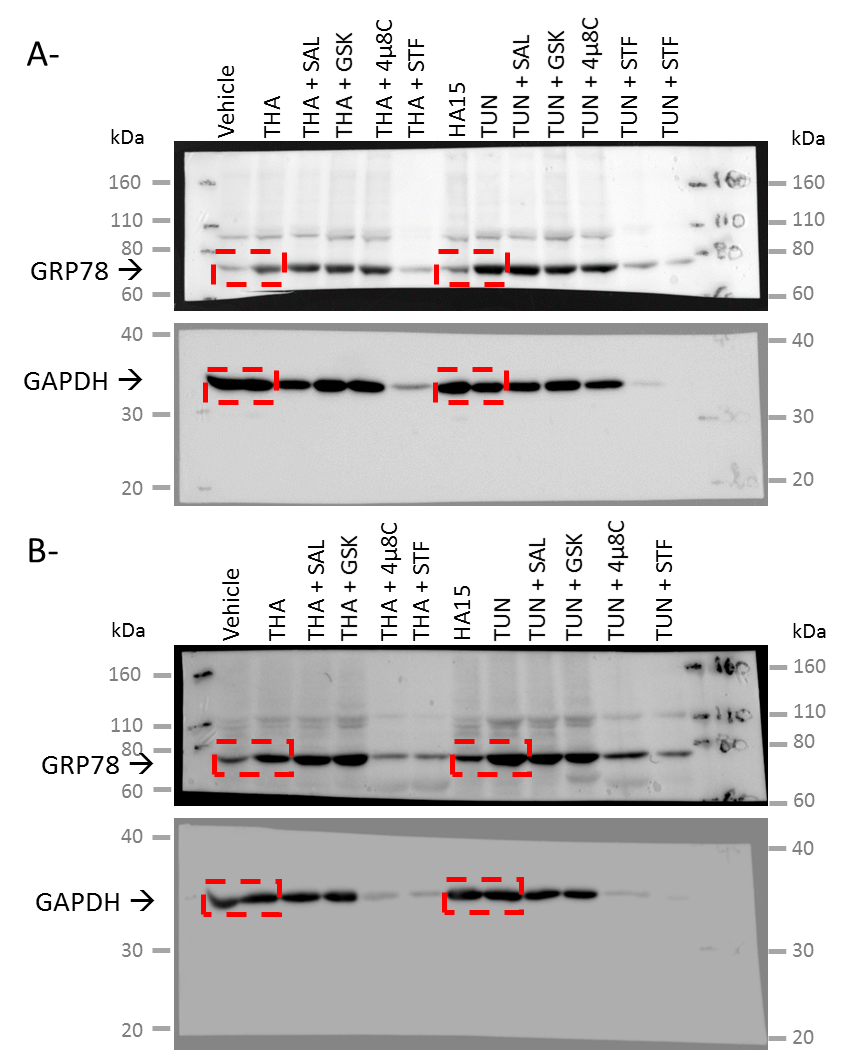


**Supplementary Figure 4.** Full images of western blots shown in Figure 3. SKOV3 cells treated with Paclitaxel (0, 1, 10 and 100 nM). A- Red dotted lines indicate the cropping locations. Ptx: Paclitaxel; n1: first experiment; n2: second experiment. B- Pictures of membranes C- Original images at different exposition time. Red dotted lines indicate the cropping locations used for images shown in A-.

A-


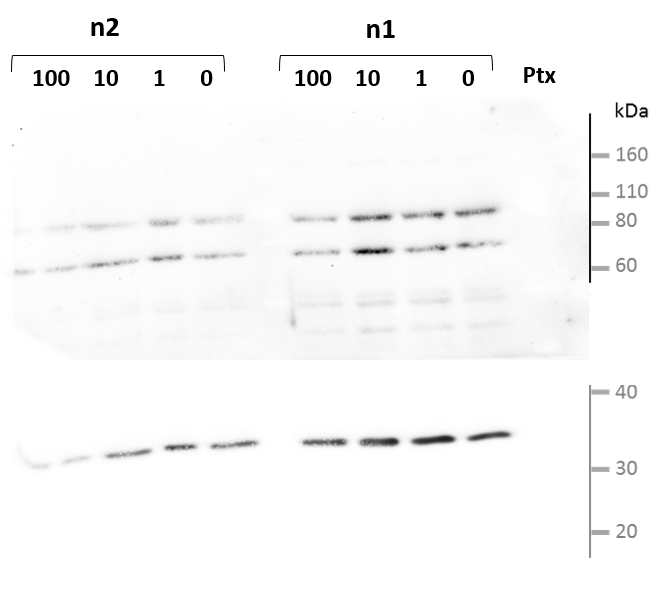

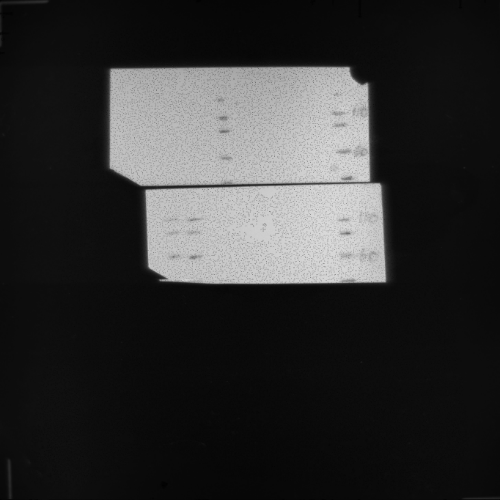


B-

GRP78 membrane

**← GAPDH**

**← GRP78**

**
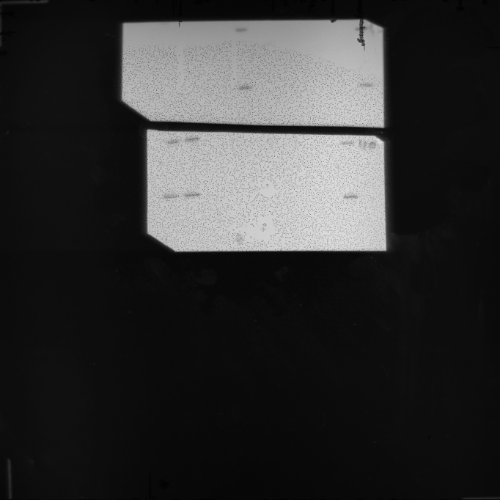
**

GAPDH membrane

C-

Original image, GRP78 membrane, 2m19s


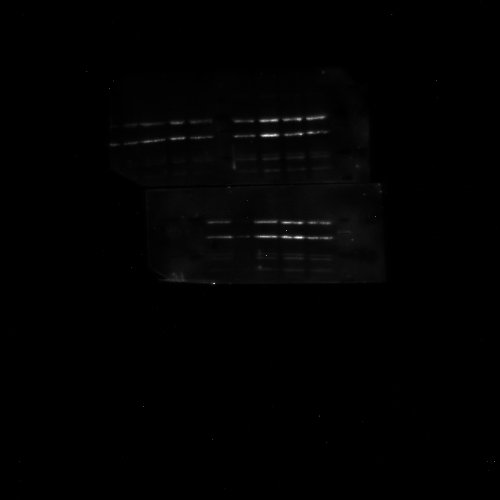


Original image, GRP78 membrane, 1m49s

**
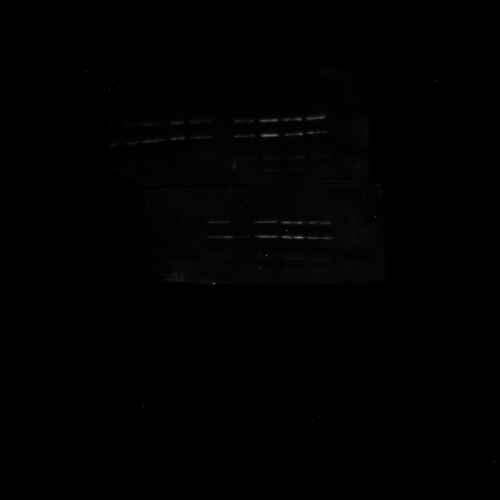
**

Original image, GAPDH membrane, 30s

**
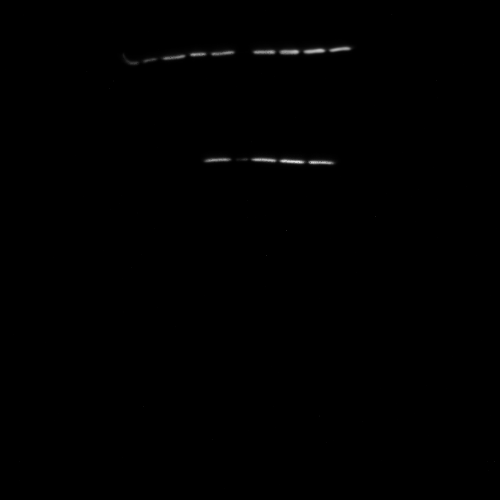
**

Original image, GAPDH membrane, 15s

**
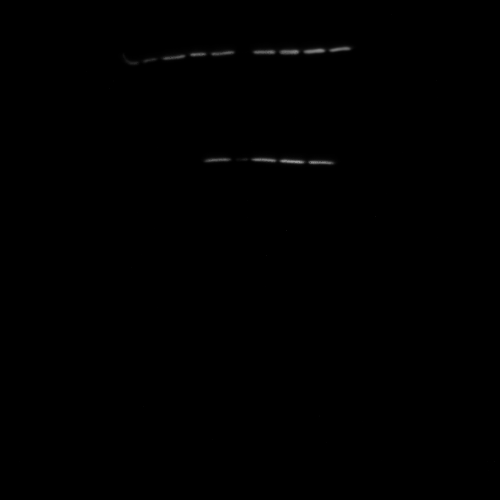
**

**Supplementary Figure 5.** Full image of zymogram shown in Figure 5. SKOV3-Green (lines 2, 5 and 9), SKOV3-Red (lines 3, 6, and 10), SKOV3-M (lines 4, 7 and 11) and conditioned medium of MCF7 (line 1, marker). Red dotted lines indicate the cropping locations. N1: first experiment; n2: second experiment; n3: third experiment.

**
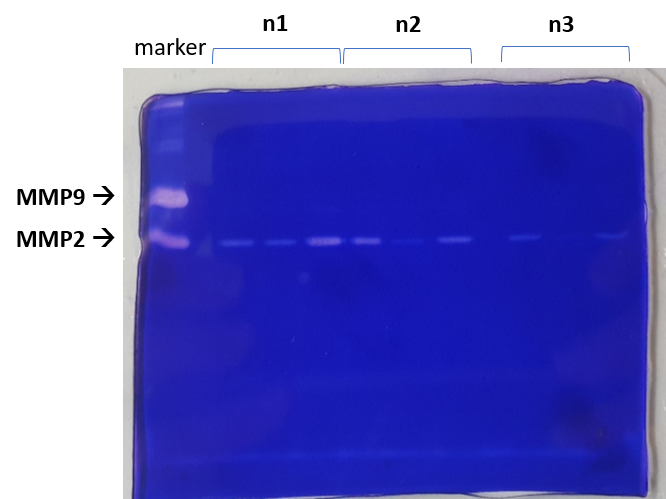
**
